# Identification of Enolase 1 and Thrombospondin-1 as serum biomarkers in HBV hepatic fibrosis by proteomics

**DOI:** 10.1186/1477-5956-11-30

**Published:** 2013-07-11

**Authors:** Bin Zhang, Zi Wang, Bin Deng, Xiaoqiong Wu, Jing Liu, Xueping Feng

**Affiliations:** 1 Department of Histology and Embryology, Xiangya School of Medicine, Central South University, 172 Tongzipo Road, Changsha, Hunan 410013, People’s Republic of China; 2 Molecular Biology Research Center, Xiangya School of Medicine, Central South University, 87, Xiangya Road, Changsha, Hunan 410008, People’s Republic of China; 3 Department of Cardiology, Xiangya Hospital, Central South University, 87, Xiangya Road, Changsha, Hunan 410008, People’s Republic of China; 4 Department of Anatomy and Neurobiology, Xiangya School of Medicine, Central South University, 172 Tongzipo Road, Changsha, Hunan 410013, People’s Republic of China; 5 Institute of Medical Sciences, Xiangya Hospital, Central South University, 87, Xiangya Road, Changsha, Hunan 410008, People’s Republic of China

**Keywords:** Hepatic fibrosis, Serum proteomics, HBV, Biomarker, Enolase-1, TSP-1

## Abstract

Hepatic fibrosis is an inevitable process in the progression of chronic HBV infection to hepatic cirrhosis, but its detailed mechanism is still unknown. Clinic serum biomarkers of HBV hepatic cirrhosis were scanned by proteomic methods. We used two-dimensional electrophoresis (2-DE) and Matrix-Assisted Laser Desorption/Ionization Time of Flight Mass Spectrometry (MALDI-TOF-MS) to separate and identify the proteins which were differentially expressed in the serum of patients with hepatic fibrosis compared to HBV carriers. We identified 27 differentially expressed proteins, of which 19 proteins were up-regulated and 8 proteins were down-regulated in the serum of patients with hepatic fibrosis compared to HBV carriers. The expression level of enolase-1 (α-enolase) was decreased while the level of thrombospondin-1 (TSP-1) increased in the serum of patients with hepatic fibrosis by western blot. Enolase-1 and TSP-1 may be useful as biomarkers for the clinic diagnosis of hepatic fibrosis, but further study is necessary.

## Introduction

Most patients with chronic HBV infection will develop hepatic cirrhosis. During the process of cirrhosis, hepatic fibrosis is an inevitable process in the development of chronic HBV infection to hepatic cirrhosis [1,2]. Hepatic fibrosis is an early event in cirrhosis in patients with HBV infection. The gold standards for the diagnosis of hepatic fibrosis are pathological staging and classification [3]. However, biopsy of hepatic fibrosis in patients is limited by sampling error, poor compliance of patients, and difficulty in drawing the materials from live tissue [4,5]. Methods which will achieve early diagnosis of hepatic fibrosis are still unknown.

The use of serum biomarkers for diagnosis of hepatic fibrosis has many advantages including being noninvasive, quick to acquire data, and provides confirmation of hepatic fibrosis in HBV infected patients faster [6]. However, there are no suitable serum biomarkers known that can serve as a reliable diagnostic biomarker for hepatic fibrosis.

Proteomics is an effective method to obtain high-flux protein data useful for identifying biomarkers [7]. In this study, in an effort to identify a serum biomarker of hepatic fibrosis, we used 2-DE and MALDI-TOF-MS to compare proteins which were differentially expressed in the serum of patients with hepatic fibrosis compared to HBV carriers. We identified two proteins, enolase-1 (α-enolase) and Thrombospondin-1 (TSP-1), which were differentially expressed and were chosen for further study. Our study suggests enolase-1 and TSP-1 play important roles in the development of hepatic fibrosis.

## Materials and methods

### Serum samples

Serum samples include 4 from patients with HBV hepatic fibrosis and another 4 from HBV carriers. All samples were from the First Xiangya Hospital of Central South University during the period of 2008.1 to 2010.12. All patients with HBV hepatic fibrosis were confirmed by pathological biopsy by two pathologists. The diagnostic criteria to classify a patient as an HBV carrier were as follows: HBsAg (+), HBV DNA (+), HBeAg or anti-HBeAb (+), and with normal level of ALT and AST, and no abnormal histological biopsy. All serum samples were drawn using a K3 EDTA anticoagulation tube and stored at 4°C in the freezer after centrifuging at 2500 rpm × 15 min The blood plasma of the upper layer was taken using suction and the samples were stored at -80°C.

### Reagents

Micro Bio Spin column and protein assay reagents were from Bio-Rad (Hercules, CA). Mouse anti-human GAPDH antibody was purchased from Sigma Co. Ltd., Mouse anti-human enolase 1, TSP-1 and secondary antibody were purchased from Santa Cruz Co. Ltd. ProteoExtract^®^ Subcellular Proteome Extraction Kit was purchased from MERCK Co. Ltd.

### Methods

#### Serum sample preparation

All serum samples were transported on ice and centrifuged at 3000 g for 15 min at 4°C. The supernatants were stored at -80°C until further analysis. Serum samples from two patients with HBV hepatic fibrosis and two HBV carriers were used for the proteomics study. The serum samples from the other four patients with HBV hepatic fibrosis and four HBV carriers were used for the western blotting study.

#### 2-DE electrophoresis

Albumin was first removed from the serum samples prior to electrophoresis. 60 μl serum were added into 180 μl combined buffer for 15 min. And Albumin were separated by adsorbing into the spin column. Collecting the supernatant fluid and used for 2-DE. The operation of 2-DE was conducted according to the ProteoExtract^®^ Subcellular Proteome Extraction Kit manual. Serum samples of 1000 μg were mixed with rehydration solution [7 mol/L urea, 2 mol/L thiourea, 0.2% DTT, 0.5% (v/v) pH3-10 IPG buffer, and a trace of bromophenol blue in a total volume of 450 μl]. Then, the samples were applied to IPG strips (pH 3-10L, 24 cm) by 14 h rehydration at 30 V, 1 h at 500 V, 1 h at 1000 V and 8.5 h at 8000 V to give a total of 68 kVh on IPGphor. Focused IPG strips were equilibrated for 15 min in a solution of 6 mol/L urea, 2% SDS, 30% glycerol, 50 mmol/L Tris–HCl (pH 8.8), and 1% DTT, and then a further 15 min in the same solution except that DTT was replaced by 2.5% iodoacetamide. IPG strips were removed after isoelectric focusing and placed into 10 ml of equilibrium A fluid [50 mmol/L Tris–HCl pH8.8, 6 mol/L urea, 30% glycerol, 1% SDS, 0.2% DTT, trace quantities of bromophenol blue] and then 10 ml equilibrium B fluid [50 mmol/L Tris–HCl pH8.8, 6mol/L urea, 30% glycerol, 1% SDS, 3% iodoacetamide, trace quantities of bromophenol blue], and equilibrated for 15 min each. SDS-PAGE was performed on an Ettan DALT II system. The protein samples were degenerated for 3 min in 100°C boiling water and then the samples were added into the top hole of a 12.5% PAGE separation gel on the Ettan DALT II system. Electrophoresis was performed at 2.5W for 30 min and then 17W until the samples reached the bottom margin of the PAGE separation gel. G-250 was used to visualize the protein spots in the 2-DE gels.

#### Image analysis

2-DE gels were scanned by an Images Scanner used MagicScan software (Amersham Biosciences) and analyzed by the PDQuest system (Bio-Rad Laboratories). Three separate gels were prepared for each cell line. The gel spot pattern of each gel was summarized in a standard gel after spot matching. Thus, we obtained one standard gel for each sample. The intensities of the spots were quantified by calculation of spot volume after normalization of the image using the total spot volume normalization method multiplied by the total area of all the spots. Proteins were identified as being differentially expressed when the spot intensity showed a difference of 2-fold. Significant differences were determined by the Student’s test with a set value of P < 0.05.

#### Protein identification by MS

All the differential spots were excised from the stained 2-DE gels using a punch. An aliquot of 1 ml 50% methyl cyanide and 50 mol/L (NH_4_)_2_CO_3_ were used to decolorize gels at 37°C for 30 min in a water bath. The tryptic peptide (0.1 μg/ml) was mixed with an R-cyano-4-hydroxycinnamic acid matrix solution. One microliter of the mixture was analyzed with a Voyager System DE-STR 4307 MALDI-TOF Mass Spectrometer (ABI) to obtain a peptide mass fingerprint (PMF). All the data were searched in a peptide mass fingerprint map database and Mascot Distiller was used to obtain the monoisotopic peak list from the raw mass spectrometry files.

From the tandem mass spectrometry database query, the peptide sequence tag (PKL) format file that was generated from MS/MS was imported into the Mascot search engine (http://www.matrixscience.com) with a MS/MS tolerance of (±0.3 Da) to search against SwissProt 2012_05 database (536,029 sequences; 190, 235, 160 residues). The parameters were selected as follows:Enzyme: Trypsin; Variable modifications: Carbamidomethyl (C), Oxidation (M); Mass values: Monoisotopic; Protein Mass: Unrestricted; Peptide Mass Tolerance: ± 0.3 Da; Fragment Mass Tolerance: ± 0.3 Da. Those proteins with scores >61 were identified as being different proteins (p < 0.05).

#### Western blotting

The final protein concentration was measured by the Bradford method [8]. A 40 μg serum protein sample was used for the gel electrophoresis in a 12% PAGE separation gel on an Ettan DALT II system. Then the samples were electrotransferred onto a PVDF membrane. Blots were blocked with 5% nonfat dry milk for 2 h at room temperature, and then incubated with primary anti-enolase 1, anti-TSP-1 for 2 h at room temperature, followed by incubation with a horseradish peroxidase-conjugated secondary antibody for 1 h at room temperature. The signal was visualized with an enhanced chemiluminescence detection reagent and quantitated by densitometry using an ImageQuant image analysis system (Storm Optical Scanner, Molecular Dynamics). The mouse anti-enolase 1 (1:4000, Sigma) and anti-TSP-1(1:4000, Sigma) were detected simultaneously as loading controls.

## Results

### Establishment of 2-DE maps of HBV carrier and hepatic fibrosis

**Figure 1:**
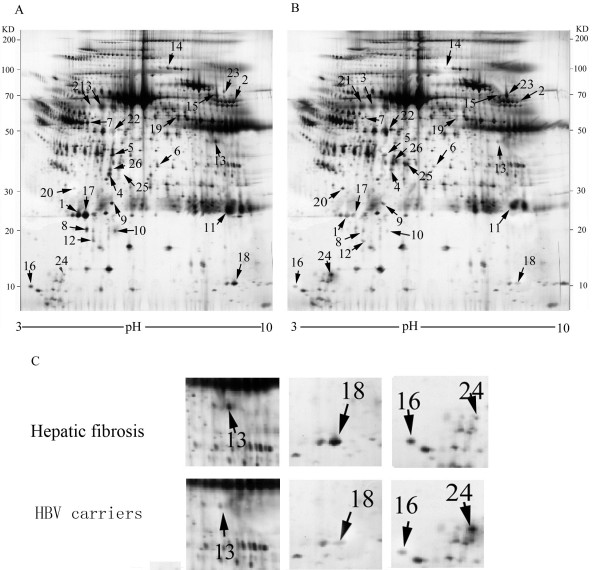
**Representative serum 2-DE maps of HBV hepatic fibrosis and HBV carriers. A**. Map of HBV hepatic fibrosis. **B**. Map of HBV carriers. The protein spots marked with arrows are the twenty-seven differential protein spots identified by MS. **C**. Close-up of the region of the gels showing some of the proteins differentially expressed between HBV hepatic fibrosis patients and HBV carriers.

We obtained 38 differentially expressed spots with ≥2 fold difference between HBV carriers and hepatic fibrosis patients. The spots were detected by MS and 27 differentially expressed proteins were determined (Figure 1). Figure 1C provides a closer view of the maps of some of the differentially expressed proteins.

**Figure 2:**
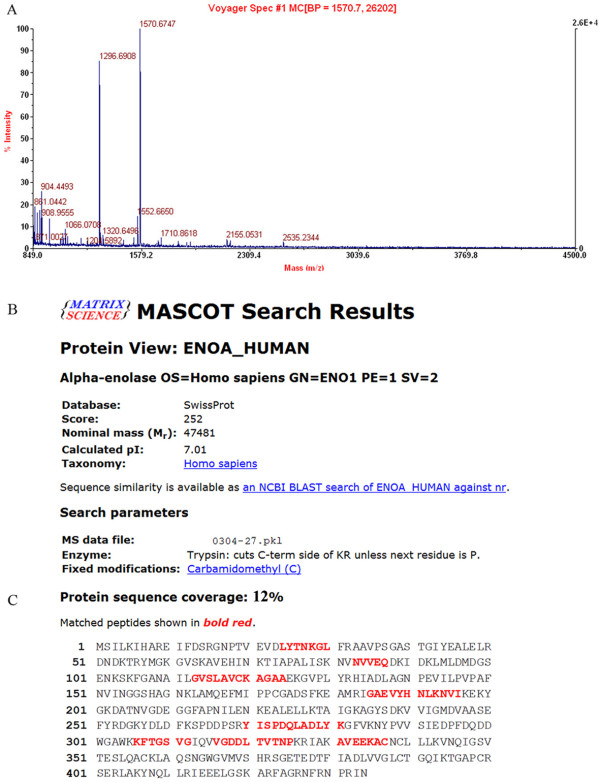
**MALDI-TOF-MS analysis of differential protein spot 27. A**. The MALDI-TOF-MS mass spectrum of spot 27; **B**. Mascot search results of spot 27, which was identified as enolase-1 according to the matched peaks; **C**. The protein sequence coverage and matched peptides of enolase-1.

2.2 MS identification of differentially expressed proteins. A total of 27 differentially expressed proteins were identified by MALDI-TOF-MS (Table 1). The data were searched in the SwissProt 2012_05 (536,029 sequences; 190,235,160 residues) database using the Mascot search engine. Enolase-1 was identified as one of the differentially expressed proteins and its mass finger print and Mascot search results are shown in Figure 2. The identified proteins are involved in cell growth, receptor binding, metastasis, blood coagulation, calcium ion binding, and DNA binding as well as some were enzymes and biomarkers (Table 1).

**Table 1:** Differential expressed proteins identified by MALDI-TOF-MS between serum of HBV hepatic fibrosis and HBV carriers

**Spot**	**Accession number**	**Protein name**	**Mass weight**	**pI**	**Sequence coverage (%)**	**Scores**	Ratio^*^	**Function**
1	P02771	Alpha-fetoprotein	69500	5.31	19	203	↑2.01	biomarker
2	P08833	Insulin-like growth factor-binding protein 1	33200	4.9	23	262	↑2.01	Cell growth
3	P02741	C-reactive protein	23760	5.12	23	570	↑2.02	calcium binding
4	P19838	Nuclear factor NF-kappa-B p105 subunit	105356	6.09	18	186	↑2.02	DNA binding
5	P2671	Fibrinogen alpha chain	66818	7.65	8	298	↑2.03	receptor binding
6	Q03135	Caveolin-1	20472	6.95	9	78	↑2.03	enzyme activity
7	P05121	Plasminogen activator inhibitor 1	48577	6.77	12	138	↑2.11	blood coagulation
8	P00505	Aspartate aminotransferase	47476	4.87	19	110	↑2.26	catalytic activity
9	P01137	Transforming growth factor beta-1	40412	5.86	12	188	↑2.29	Cell growth
10	P04271	Protein S100-B	10713	4.43	8	154	↑2.31	calcium binding
11	P24298	Alanine aminotransferase 1	54637	5.11	13	908	↑2.89	catalytic activity
12	Q14116	Interleukin-18	21326	5.23	27	392	↑2.97	signal transducer
13	P01375	Tumor necrosis factor	25644	5.43	18	152	↑3.01	apoptotic process
14	P08253	Matrix metallopeptidase 2	73882	6.57	30	150	↑3.01	metastasis
15	P01579	Interferon gamma	19348	5.67	16	335	↑3.21	receptor binding
16	P01034	Cystatin-C	13120	7.11	13	262	↑3.57	enzyme activity
17	P05187	Alkaline phosphatase	57954	6.14	16	362	↑3.66	hydrolase activity,
18	P01033	Metalloproteinase inhibitor 1	23171	5.28	4	74	↑4.21	metastasis
19	P07996	Thrombospondin-1	25695	6.67	6	166	↑7.56	blood coagulation
20	P17936	Insulin-like growth factor-binding protein 3	31674	5.03	8	304	↓2.17	Cell growth
21	P37231	Peroxisome proliferator-activated receptor gamma	57620	5.23	25	946	↓2.72	DNA binding
22	P04180	Phosphatidylcholine-sterol acyltransferase	49578	5.68	17	541	↓2.88	catalytic activity
23	P06276	Cholinesterase	68418	6.92	16	765	↓3.12	catalytic activity
24	P02652	Apolipoprotein A-II	12520	4.74	28	552	↓3.22	receptor binding
25	O46409	Apolipoprotein A-IV	43294	5.74	26	550	↓3.57	receptor binding
26	Q6Q788	Apolipoprotein A-V	41213	4.97	13	394	↓3.59	receptor binding
27	P19875	Enolase-1	47481	7.01	12	252	↓5.09	DNA binding

^*^ Hepatic fibrosis vs HBV carriers.

**Figure 3:**
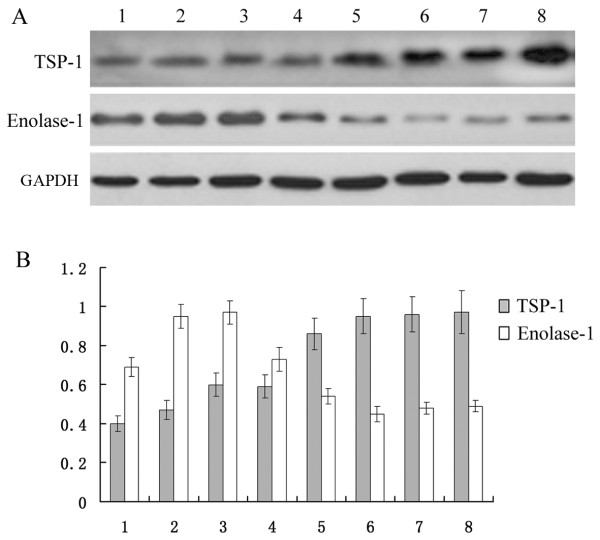
**Western blots of Enolase-1 and TSP-1 in the serum of hepatic fibrosis patients and HBV carriers. A**, Western blot analysis showing changes in the expression level of enolase 1 and TSP-1 in the serum of HBV carriers and hepatic fibrosis patients. **B**, Histogram shows the expression level of the proteins in the serum of HBV carriers and hepatic fibrosis patients as analyzed by Bandleader 3.0 software. Note: bands 1, 2, 3, and 4 are from HBV carriers and bands 5, 6, 7, and 8 are from HBV hepatic fibrosis patients.

2.3 Verification of differentially expression proteins by western blot. The two proteins, enolase-1 and thrombospondin-1(TSP-1) were detected by western blot in the serum of hepatic fibrosis patients and HBV carriers. Enolase-1 was at least 5.09-fold down-regulated in hepatic fibrosis patients compared to HBV carriers and TSP-1 was at least 7.56-fold up-regulated in hepatic fibrosis patients compared to HBV carriers. Enolase-1 and TSP-1 were detected in eight cases (four hepatic fibrosis patients and four HBV carriers). The results showed that, compared to HBV carriers, expression of enolase-1 was down regulated in the serum of patents with hepatic fibrosis, while expression of TSP-1 was up regulated in the serum of patients with hepatic fibrosis (Figure 3). The results of western blot confirmed the results from the proteomics study.

## Discussion

Hepatic fibrosis is an inevitable process in the transformation of chronic HBV infection to hepatic cirrhosis, but its detailed mechanism is still unknown. Fontana et al. retrospectively analyzed the clinical progression of serum hepatic fibrosis markers obtained from currently laboratory methods and discovered that these clinical test methods can only reliably recognize advanced fibrosis, and cannot accurately identify the degree of fibrosis present during the early and middle portions of its progression [9]. Therefore, identification of HBV hepatic fibrosis biomarkers is of great significance for early diagnosis and prevention of fibrosis.

Serum proteomics studies serum proteins which are readily available. However, because of proteome analysis restrictions for sample size, highly abundant proteins often make it difficult to separation and identify less abundant proteins, an issue which is particularly evident in the proteomic analyses of serum [10]. Thus, it is necessary to first remove the interfering high abundance proteins prior to serum proteome analysis. In this study, we removed the albumin which improves the detection rate of low abundance proteins with good reproducibility. A total of 27 differentially expressed genes were found in patients with HBV hepatic fibrosis compared to the plasma of HBV carriers, of which 19 were up-regulated and 8 were down-regulated in the serum of patients with HBV hepatic fibrosis.

As one of the key enzymes of glycolysis, enolase-1 (α-enolase) widely exists in many tissues and its expression varies with cellular pathological physiology, metabolism, inflammation, and the state of cell development [11,12]. Enolase-1 also plays an important role in cell energy metabolism [13]. Enolase-1 is expressed at the cell surface where it promotes cancer invasion, and is subjected to a specific array of post-translational modifications, namely acetylation, methylation and phosphorylation. Enolase-1 binds plasminogen at the cell surface, enhancing local plasmin production and monocyte migration through epithelial monolayers, and promoting matrix degradation. These data suggest an important mechanism of inflammatory cell invasion is mediated by increased cell-surface expression of enolase-1 [14]. Both enolase-1 over expression and its post-translational modifications could be of diagnostic and prognostic value in cancer [15]. Takashima et al. analyzed the hepatic tissue of patients with hepatitis b virus-related hepatocellular carcinoma (HCC) by proteomics analysis and found that expression of enolase-1 was enhanced, which is particularly apparent in poorly-differentiated HCC [16]. Enolase-1 acts as a central element in colon cancer susceptibility and protein biosynthesis [17]. However there are no reports about enolase-1 and hepatic fibrosis. Our experimental results indicated that the expression level of enolase-1 in the serum of patients with HBV hepatic fibrosis was significantly higher than that in HBV carriers. Its change of concentration in the blood may reflect the degree of hepatic fibrosis suggesting that enolase-1 can be used as a serum marker for the prediction of hepatic fibrosis.

Thrombospondin-1 (TSP-1) is a glycoprotein with a molecular weight of 450 kDa, and is a major component of platelets [18]. It is involved in angiogenesis and inflammation and the effects of TSP-1 have been studied in numerous preclinical tumor models. Many normal cells, including endothelial cells, fibroblasts, adipocytes, smooth muscle cells, monocytes, and macrophages were all founded to secrete TSP-1 [19,20]. TSP-1 binds to the protein components of the extracellular matrix such as fibronectin. TSP-1 is also expressed in glomerulopathies and is considered an early marker of inflammation and fibrosis [21].

TSP-1 can bind to various receptors and functions in regulating cell proliferation, proteases and the activity of transforming growth factor beta-1 (TGF-β1) [22,23]. As an activator of TGF-β1, TSP-1 modulates the functions of TGF-β1 in cardiovascular diseases, atherosclerosis, and obesity. In addition, TSP-1 is selectively expressed in the infracted border suggesting that TSP-1 might inhibit the expansion of inflammation by activating TGF-β1 [24]. TSP1 is also a major endogenous activator of TGF-β1 in experimental inflammatory glomerular disease. Regulation of TSP-1 with regards to its influence on TGF-β1 activity may be one of the causes of fibrosis, as TGF-β1 positively regulates HCV RNA replication which is likely manifested in the liver fibrosis associated with hepatitis C infection [25]. It has been reported that TSP-1 is closely related to renal fibrosis in which TSP-1 is highly expressed [26]. In studies of hepatic fibrosis, TSP-1 is found to be highly expressed. TSP-1 might play an additional role in liver fibrogenesis by stimulating angiogenesis and could be a potential target to prevent fibrogenesis in chronic inflammatory diseases of the liver [27].

NF-κB is a transcription factor present in almost all animal cell types. It is involved in many biological processes such as inflammation, immunity, cell differentiation, cell growth, tumorigenesis and apoptosis [28]. Studies indicate that NF-κB and TSP-1 together, modulated by the expression of the androgen receptor, exert antitumor effects in prostate cancer [29]. In the tumor microenvironment, TSP-1 has been shown to suppress tumor growth by inhibiting angiogenesis and activating TGF-β1. NF-κB mRNA expression and activity were significantly enhanced by proteinuria-loading and were synchronized with high expression of TSP-1, and TGF-β1 mRNA in the kidney [30]. We found that expression of TSP-1, TGF-β1, and NF-κB in patients with HBV hepatic fibrosis were all significantly higher than that in the serum of HBV carriers, suggesting that TSP-1, TGF-β1, and NF-κB play important roles in the process of hepatic fibrosis. The detailed mechanisms involved in the interplay between TSP-1, TGF-β1, NF-κB and hepatic fibrosis require further study.

In conclusion, using a proteomics method, we obtained 27 differentially expressed proteins by comparing the serum of HBV carriers and hepatic fibrosis patients. The 27 proteins identified by proteomic methods are involved in cell growth, receptor binding, metastasis, blood coagulation, calcium ion binding, DNA binding, as well as being biomarkers and enzymes. Additional work is required to understand the relationship between these proteins and hepatic fibrosis. The results indicated that TSP-1, TGF-β1, NF-κB, and enolase-1 likely play important roles in the process of hepatic fibrosis. Although the levels of enolase-1 and TSP-1 in the serum of HBV carriers and hepatic fibrosis patients can be detected, the relationship between the degree of liver fibrosis and their expression needs further study.

## Abbreviations

HBV: Hepatitis B virus; MS: Mass spectrometry; 2-DE: Two-dimensional electrophoresis; MALDI-TOF-MS: Matrix-Assisted Laser Desorption/Ionization Time of Flight Mass Spectrometry; TSP-1: Thrombospondin-1; HCC: Hepatocellular carcinoma.

## Competing interests

The authors declare that they have no competing interests.

## Authors’ contributions

BZ, JL and XF designed the study. BZ, BD, XW, JL and XF performed the work. BZ, WZ, BD, and XW interpreted the data. BZ, JL and XF wrote the manuscript. BZ, WZ, BD, XW, JL and XF approved the manuscript. All authors read and approved the final manuscript.

## References

[B1] Piao RL Brigstock DR Clinical significance of connective tissue growth factor in hepatitis B virus-induced hepatic fibrosis World J Gastroenterol 2012 18 18 2280 2286 22611323 10.3748/wjg.v18.i18.2280 PMC3351780

[B2] Sun HQ Zhang JY Increased Th17 cells contribute to disease progression in patients with HBV-associated liver cirrhosis J Viral Hepat 2012 19 6 396 403 22571901 10.1111/j.1365-2893.2011.01561.x

[B3] Becker L Salameh W Sferruzza A Validation of hepascore, compared with simple indices of fibrosis, in patients with chronic hepatitis C virus infection in United States Clin Gastroenterol Hepatol 2009 7 6 696 701 19514117 10.1016/j.cgh.2009.01.010

[B4] Germani G Hytiroglou P Assessment of fibrosis and cirrhosis in liver biopsies: an update Semin Liver Dis 2011 31 1 82 90 21344353 10.1055/s-0031-1272836

[B5] Bioulac-Sage P Primary biliary cirrhosis: a new histological staging and grading system proposed by Japanese authors Clin Res Hepatol Gastroenterol 2011 35 5 333 335 21497151 10.1016/j.clinre.2011.03.001

[B6] Nakanuma Y Zen Y Application of a new histological staging and grading system for primary biliary cirrhosis to liver biopsy specimens: Interobserver agreement Pathol Int 2010 60 3 167 174 20403042 10.1111/j.1440-1827.2009.02500.x

[B7] Pham TV Piersma SR Label-free mass spectrometry-based proteomics for biomarker discovery and validation Expert Rev Mol Diagn 2012 12 4 343 359 22616700 10.1586/erm.12.31

[B8] Bradford MM A rapid and sensitive method for the quantitation of microgram quantities of protein utilizing the principle of protein-dye binding Anal Biochem 1976 72 248 254 942051 10.1016/0003-2697(76)90527-3

[B9] Fontana RJ Goodman ZD Relationship of serum fibrosis markers with liver fibrosis stage and collagen content in patients with advanced chronic hepatitis C Hepatology 2008 47 3 789 798 18175357 10.1002/hep.22099

[B10] Ray S Reddy PJ Proteomic technologies for the identification of disease biomarkers in serum: advances and challenges ahead Proteomics 2011 11 11 2139 2161 21548090 10.1002/pmic.201000460

[B11] Gerlt JA Babbitt PC Divergent evolution in enolase superfamily: strategies for assigning functions J Biol Chem 2012 287 1 29 34 22069326 10.1074/jbc.R111.240945 PMC3249080

[B12] Butterfield DA Lange ML Multifunctional roles of enolase in Alzheimer’s disease brain: beyond altered glucose metabolism J Neurochem 2009 111 4 915 933 19780894 10.1111/j.1471-4159.2009.06397.x PMC4454338

[B13] Freeman WD Chiota NA Neuron-specific enolase correlates with other prognostic markers after cardiac arrest Neurology 2011 77 20 1856 1857 22191105

[B14] Wygrecka M Marsh LM Enolase-1 promotes plasminogen-mediated recruitment of monocytes to the acutely inflamed lung Blood 2009 113 22 5588 5598 19182206 10.1182/blood-2008-08-170837

[B15] Capello M Ferri-Borgogno S α-Enolase: a promising therapeutic and diagnostic tumor target FEBS J 2011 278 7 1064 1074 21261815 10.1111/j.1742-4658.2011.08025.x

[B16] Takashima M Kuramitsu Y Overexpression of alpha enolase in hepatitis C virus-related hepatocellular carcinoma:association with tumor progression as determined by p roteomic analysis Proteomics 2005 5 6 1686 1692 15800975 10.1002/pmic.200401022

[B17] Jiang W Li X Constructing disease-specific gene networks using pair-wise relevance metric: application to colon cancer identifies interleukin 8, desmin and enolase 1 as the central elements BMC Syst Biol 2008 2 72 18691435 10.1186/1752-0509-2-72 PMC2535780

[B18] Lawler PR Lawler J Molecular basis for the regulation of angiogenesis by thrombospondin-1 and -2 Cold Spring Harb Perspect Med 2012 2 5 a006627 22553494 10.1101/cshperspect.a006627 PMC3331684

[B19] Wight TN Raugi GJ Light microscopic immunolocation of thrombospondin in human tissues J Histochem Cytochem 1985 33 4 295 302 3884704 10.1177/33.4.3884704

[B20] Naganuma H Satoh E Quantification of thrombospondin-1 secretion and expression of alphavbeta3 and alpha3beta1 integrins and syndecan-1 as cell-surface receptors for thrombospondin-1 in malignant glioma cells J Neurooncol 2004 70 3 309 317 15662972 10.1007/s11060-004-9167-1

[B21] McGregor B Colon S Thrombospondin in human glomerulopathies. A marker of inflammation and early fibrosis Am J Pathol 1994 144 6 1281 1287 7515560 PMC1887455

[B22] Ioachim E Damala K Thrombospondin-1 expression in breast cancer: prognostic significance and association with p53 alterations, tumour angiogenesis and extracellular matrix components Histol Histopathol 2012 27 2 209 216 22207555 10.14670/HH-27.209

[B23] Rojas A Meherem S The aberrant methylation of TSP1 suppresses TGF-beta1 activation in colorectal cancer Int J Cancer 2008 123 1 14 21 18425817 10.1002/ijc.23608 PMC2777657

[B24] Daniel C Wiede J Thrombospondin-1 is a major activator of TGF-beta in fibrotic renal disease in the rat in vivo Kidney Int 2004 65 2 459 468 14717916 10.1111/j.1523-1755.2004.00395.x

[B25] Presser LD Haskett A Hepatitis C virus-induced furin and thrombospondin-1 activate TGF-β1: role of TGF-β1 in HCV replication Virology 2011 412 2 284 296 21296375 10.1016/j.virol.2010.12.051 PMC3073624

[B26] Frangogiannis NG Ren G Critical role of endogenous thrombospondin-1 in preventing expansion of healing myocardial infarcts Circulation 2005 111 22 2935 2942 15927970 10.1161/CIRCULATIONAHA.104.510354

[B27] Elpek GO Gokhan GA Thrombospondin-1 expression correlates with angiogenesis in experimental cirrhosis World J Gastroenterol 2008 14 14 2213 2217 18407596 10.3748/wjg.14.2213 PMC2703847

[B28] Johnson RF Perkins ND Nuclear factor-κB, p53, and mitochondria: regulation of cellular metabolism and the Warburg effect Trends Biochem Sci 2012 37 8 317 324 22626470 10.1016/j.tibs.2012.04.002

[B29] Nelius T Filleur S Androgen receptor targets NfkappaB and TSP1 to suppress prostate tumor growth in vivo Int J Cancer 2007 121 5 999 1008 17487836 10.1002/ijc.22802 PMC2810747

[B30] El-Youssef M Mu Y Increased expression of transforming growth factor-beta1 and thrombospondin-1 in congenital hepatic fibrosis: possible role of the hepatic stellate cell J Pediatr Gastroenterol Nutr 1999 28 4 386 392 10204502 10.1097/00005176-199904000-00008

